# Dataset of the EnergyPlus model used in the assessment of natural ventilation potential through building simulation

**DOI:** 10.1016/j.dib.2021.106753

**Published:** 2021-01-15

**Authors:** N.R.M. Sakiyama, L. Mazzaferro, J.C. Carlo, T. Bejat, H. Garrecht

**Affiliations:** a Materials Testing Institute (MPA) University of Stuttgart, Pfaffenwaldring 2b, 70569 Stuttgart, Germany; b Institute for Science, Engineering and Technology (ICET), Federal University of the Jeq. and Muc. Valleys (UFVJM), R. Cruzeiro, 01 - Jardim São Paulo, 39803-371 Teófilo Otoni, Brazil; c Laboratory of Energy Efficiency in Buildings (LabEEE), Federal University of Santa Catarina (UFSC), Caixa Postal 476, 88040970 Florianópolis, Brazil; d University Grenoble Alpes, CEA, LITEN, DTS, LIPV, INES, F-38000 Grenoble, France; e Architecture and Urbanism Department (DAU), Federal University of Vicosa (UFV), Av P. H. Rolfs, 36570 900 Viçosa, Brazil

**Keywords:** Building simulation, EnergyPlus, Airflow network, Hybrid system, Natural ventilation

## Abstract

The data set compiled in this file refers to the Multizone EnergyPlus model, used in the investigations of the research article entitled "Natural ventilation potential from weather analyses and building simulation". The technical information regarding the model has been grouped into tables, which include: the general simulation settings, the properties of the building materials, the Airflow Network opening settings used in the annual investigation, in addition to the controls established in the Energy Management System (EMS) for hybrid ventilation system operation. The user behaviour, regarding the living and bedrooms occupancy schedule, is also presented in a graph. This data set is made available to the public to clarify details of the EnergyPlus model and how the hybrid operation was defined. In this way, other researchers can perform an extended analysis of the information.

## Specifications Table

**Table:** 

Subject	Engineering, Architecture
Specific subject area	Building simulation in hybrid mode: heating loads and thermal comfort assessment
Type of data	Tables Graph Text
How data were acquired	Building energy modelling – Computer simulation using EnergyPlus software
Data format	Raw
Parameters for data collection	Building energy model (input data file -.idf) created from a full-scale test passive house
Description of data collection	The model geometry was developed using SketchUp Make 2017 with OpenStudio Plugin for EnergyPlus and the computer simulation run using EnergyPlus software, version 9.1
Data source location	French National Institute for Solar Energy - INES Chambery, French Alps France 45° 38′38.5 "N, 5° 52′27.4 "E
Data accessibility	With the article EnergyPlus files (.idf): https://data.mendeley.com/datasets/rp6xy7rfhn/1
Related research article	Sakiyama NRM, Mazzaferro L, Carlo JC, Bejat T, Garrecht H. Natural ventilation potential from weather analyses and building simulation. Energy and Buildings 2021;231:110596. https://doi.org/10.1016/j.enbuild.2020.110596

## Value of the Data

• The detailed data related to the EnergyPlus model guarantees a better and deeper understanding of the building addressed in the research paper [1], grounding the study developed and providing more information to aid in reading the paper.
• Both building modelling and simulation are performed using EnergyPlus software. Different configurations/ simulation techniques could be employed based on this available data, so different studies might be compared.
• The data presented in this article can assist designers and researchers who deal with the modelling of naturally ventilated buildings, especially with Airflow Network and multizone approach.
• The use of Energy Management System (EMS) to model hybrid ventilation operation could be adopted as a reference for further research on naturally ventilated buildings.

## 1 Data Description

**Fig. 1:**
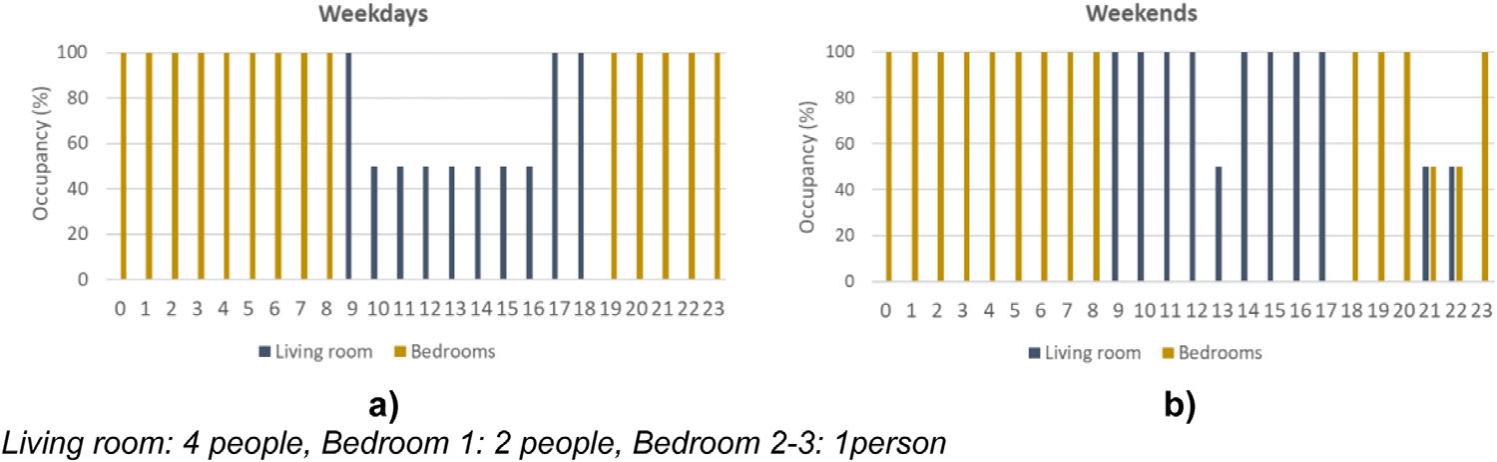
Occupancy schedule – annual simulation a=Weekdays schedule; b= Weekend schedule.

The data in this article present the input data regarding the EnergyPlus model used at the investigations addressed in the research paper. General simulation settings are summarised in Table 1, while the building materials properties are listed in Table 2. Table 3 shows the Airflow Network opening settings used in the annual investigation. Finally, the occupancy schedule, which specifies when the living and bedrooms were occupied, as well as its respective number of people, can be seen in Fig. 1.

**Table 1:** General simulation settings.

	Calibration Model (a)	Annual Analyses (b)
Run period	19^th^ till 25^th^ August	1^st^ January till 31th Dezember
Airflow simulation	Airflow Network HVACTemplate:Zone:PTHP	EMS: Airflow Network HVACTemplate: Zone:IdealLoadAirSystem
Solar distribution	FullExteriorWithReflections	
Surface convection Algorithm: Inside	TARP	
Surface convection Algorithm: Outside	DOE-2	
Heat Balance Algorithm	ConductionTransferFunction	
Monthly ground temperature (˚C)	4.5, 6.21, 9.3, 12.99, 16.28, 18.27,18.43, 16.69, 13.55, 9.86, 6.58, 4.62	
Time steps per hour	6

**Table 2:** Building material properties.

Construction type	Construction name	Material layers (outside to inside)	Thickness (m)	Conductivity (W/m-K)	Density (kg/m3)	Specific Heat (J/kg-K)	Thermal Absorptance	Solar Absorptance	Visible Absorptance
Façade	MurExt_isole	Porotherm R42 (Hollow brick)	0.425	0.115	700	986	0.9	0.6	0.6
		Plaster	0.01	0.4	1200	1000	0.9	0.6	0.6
Internal walls	Cloisons_Etage	Placo_13mm	0.013	0.25	825	1008	0.9	0.6	0.6
		Glass Wool 5cm	0.05	0.032	12	840	0.9	0.6	0.6
		Placo_13mm	0.013	0.25	825	1008	0.9	0.6	0.6
Underground walls	Mur_vs	Structural Wall	0.425	2.5	2500	1000	0.9	0.6	0.6
Underground floor slab	PB_VS	Gravel	0.05	1.4	2100	650	0.9	0.6	0.6
Underground ceiling slab	PH_VS_isole	Structural slab	0.2	2.5	2500	1000	0.9	0.6	0.6
		PolystyreneXtrude	0.16	0.027	35	1400	0.9	0.6	0.6
Ground floor slab	PH_RCD	Concrete screed	0.08	1.75	2400	880	0.9	0.6	0.6
		Compression slab	0.04	1.75	2400	880	0.9	0.6	0.6
		Hollow concrete slab	0.16	1.23	1300	648	0.9	0.6	0.6
Attic slab	PB_COMBLES_isole	Plaster					0.9	0.6	0.6
		Glass Wool Filling	0.44	0.032	12	840	0.9	0.6	0.6
		OSB Floor	0.022	0.13	640	1700	0.9	0.6	0.6
Roof	Toit	Tiles	0.015	2.2	1121	1460	0.9	0.7	0.7
		Wood Structure	0.08	0.055	265	836	0.9	0.7	0.7
		Metal Decking	0.0015	45	7680	418	0.9	0.6	0.6

The model set up is based on consolidated practices used in studies involving INES' experimental houses, and therefore does not use the EnergyPlus database. Since they are originally unoccupied, a classic family occupancy schedule was established, which would represent an extreme/worse possible scenario.

Besides, the EnergyPlus input data files (.idf) are available for download in the Mendeley repository [2], and the link can be found in the Specifications table/ Data accessibility. The files supplied include: the calibration (a) and the annual analysis (b) models, which are summarized in Table 1.

## 2 Experimental Design, Materials and Methods

The controls developed in the Energy Management System (EMS) object for the consolidation of the hybrid behaviour at the annual analyses are presented below. The operation mode was adopted in all occupied zones, exemplified here by the living room zone.

The set up enables the following changes: triggering the thermal load calculation at a temperature different from the thermostat; deactivation of the thermal load calculation only after occupancy in a room is null; hybrid control, where the local thermal prognosis is not allowed to occur together with the window opening for natural ventilation in the same time step.

**All objects in class: energymanagementsystem:sensor**

EnergyManagementSystem:Sensor,

OT_Living, !- Name

Living, !- Output:Variable or Output:Meter Index Key Name

Zone Operative Temperature; !- Output:Variable or Output:Meter Name

EnergyManagementSystem:Sensor,

Occ_Living, !- Name

Living_Occ, !- Output:Variable or Output:Meter Index Key Name

People Occupant Count; !- Output:Variable or Output:Meter Name

EnergyManagementSystem:Sensor,

Ext_Temp, !- Name

Environment, !- Output:Variable or Output:Meter Index Key Name

Site Outdoor Air Drybulb Temperature; !- Output:Variable or Output:Meter Name

EnergyManagementSystem:Sensor,

T_Living, !- Name

Living, !- Output:Variable or Output:Meter Index Key Name

Zone Mean Air Temperature; !- Output:Variable or Output:Meter Name

EnergyManagementSystem:Sensor,

Heat_Living, !- Name

Heat_Living, !- Output:Variable or Output:Meter Index Key Name

Schedule Value; !- Output:Variable or Output:Meter Name

**All objects in class: energymanagementsystem:Actuator**

EnergyManagementSystem:Actuator,

HeaterControl_Living, !- Name

Heat_Living, !- Actuated Component Unique Name

Schedule:Constant, !- Actuated Component Type

Schedule Value; !- Actuated Component Control Type

EnergyManagementSystem:Actuator,

NVControl_Living, !- Name

NV_Living, !- Actuated Component Unique Name

Schedule:Constant, !- Actuated Component Type

Schedule Value; !- Actuated Component Control Type

**All objects in class: energymanagementsystem:programcallingmanager**

EnergyManagementSystem:ProgramCallingManager,

HybridControl, !- Name

BeginTimestepBeforePredictor, !- EnergyPlus Model Calling Point

Hyb_Living, !- Program Name 1

**Table 3:** AFN opening settings – annual investigation.

	Opening name/ orientation	U-factor (W/m²K)	Solar Heat Gain Coefficient	Opening Factor	Ventilation Control Mode	Discharge Coefficient	Temp. set point	Ventilation Availability schedule
External Windows	Cellar_N	1.68	0.24	0.75	Temp.	0.50	20	EMS
	Living_E	1.4	0.21	0.75	Temp.	0.50	20	EMS
	Hall_E	1.4	0.21	0.75	Temp.	0.50	20	EMS
	Living_S	1.28	0.47	0.75	Temp.	0.50	20	EMS
	Bedroom3_S	1.34	0.39	0.75	Temp.	0.50	20	EMS
	Stairs_N	1.32	0.21	0.75	Temp.	0.50	20	EMS
Internal Doors		-	-	-	NoVent	0.55	-	-
Horizontal Opening (Stair case)		-	-	1	Constant	0.2	-	-

**All objects in class: energymanagementsystem:program**

EnergyManagementSystem:Program,

Hyb_Living, !- Name

SET Temp_Heat = T_Living <= 19, !- Program Line 1

IF ((Occ_Living > 0) && (Temp_Heat ==1)), !- Program Line 2

SET HeaterControl_Living =1, !- A4

SET NVControl_Living = 0,!- A5

ELSEIF ((Occ_Living > 0) && (Heat_Living >0)), !- A6

SET HeaterControl_Living = 1, !- A7

SET NVControl_Living = 0,!- A8

ELSEIF (Occ_Living > 0), !- A9

IF ((Ext_Temp<T_Living) && (Ext_Temp>20)), !- A10

SET HeaterControl_Living = 0, !- A11

SET NVControl_Living = 1,!- A12

ELSEIF ((Ext_Temp>T_Living) && (Ext_Temp>20)), !- A13

SET HeaterControl_Living = 0, !- A14

SET NVControl_Living = 0,!- A15

ELSEIF (Ext_Temp<20), !- A16

SET HeaterControl_Living = 0, !- A17

SET NVControl_Living = 0,!- A18

ENDIF, !- A19

ELSEIF (Occ_Living == 0),!- A20

SET HeaterControl_Living = 0, !- A21

SET NVControl_Living = 0,!- A22

ENDIF; !- A23

## CRediT Author Statement

**Nayara R. M. Sakiyama:** Conceptualization, Methodology, Software, Data-curation, Formal analysis, Investigation, Writing-Original draft preparation; **Leonardo Mazzaferro:** Software, Visualization, Validation, Writing-Reviewing and Editing; **Joyce C. Carlo:** Supervision; **Timea Bejat:** Resources; **Harald Garrecht:** Project administration.

## Declaration of Competing Interest

The authors declare that they have no known competing financial interests or personal relationships which have, or could be perceived to have, influenced the work reported in this article.

## Appendix Supplementary materials

Image, application 1

Image, application 2

Image, application 3

Image, application 4
